# Endourological Evaluation and Management of Leukoplakia of the Renal Pelvis

**DOI:** 10.1155/DTE.2.167

**Published:** 1996

**Authors:** Hidehiro Kakizaki, Katsuya Nonomura, Tomohiko Koyanagi, Masami Nantani, Kotaro Taniguchi, Tadashi Matsuno

**Affiliations:** 1 Department of Urology Hokkaido University School of Medicine Sapporo 060 Japan; 2 Hokkaido Memorial Hospital of Urology Sapporo Japan

## Abstract

Since August 1989, we have seen 4 patients with leukoplakia of the renal pelvis associated with a longstanding renal stone. In 2 of them, excretory or retrograde pyelography revealed multiple filling defects in the left renal pelvis as well as a renal stone, although urine cytological examination was negative. One of the other 2 patients underwent extracorporeal shock wave lithotripsy (ESWL) for the renal stone, but this was not followed by the passage of stone fragments. The renal stone in the remaining patient was associated with staghorn calculi. For stone extraction as well as endoscopic evaluation of the intrapelvic lesion, percutaneous nephroscopy was performed. A small to large amount of tissue-like white debris in sheets characteristic of leukoplakia was found in the renal pelvis with stones embedded in it and was removed directly by forceps or suction and then by irrigating with saline. We propose that 1) the endourological approach should be recommended for patients with renal pelvic lesions associated with longstanding renal stones or for patients who show difficulty in passing stone fragments after ESWL and 2) this entity of leukoplakia should be kept in mind for the differential diagnosis of renal pelvic lesions associated with renal stones.


*Diagnostic and Therapeutic Endoscopy*, 1996, Vol. 2, pp. 167–174
Reprints available directly from the publisher
Photocopying permitted by license only

© 1996 OPA (Overseas Publishers Association)
Amsterdam B.V. Published in The Netherlands
by Harwood Academic Publishers GmbH
Printed in Singapore

### Endourological Evaluation and Management of Leukoplakia of the Renal Pelvis

HIDEHIRO KAKIZAKI, KATSUYA NONOMURA, TOMOHIKO KOYANAGI, MASAMI NANTANI, KOTARO TANIGUCHI, and TADASHI MATSUNO

*Department of Urology, Hokkaido University School of Medicine (H.K., K.N., T.K.) and Hokkaido Memorial Hospital of Urology (M.N., K.T., T.M.). Sapporo, Japan*

*(Received February 24, 1995; in final form July 20, 1995)*

Since August 1989, we have seen 4 patients with leukoplakia of the renal pelvis associated with a longstanding renal stone. In 2 of them, excretory or retrograde pyelography revealed multiple filling defects in the left renal pelvis as well as a renal stone, although urine cytological examination was negative. One of the other 2 patients underwent extracorporeal shock wave lithotripsy (ESWL) for the renal stone, but this was not followed by the passage of stone fragments. The renal stone in the remaining patient was associated with staghorn calculi. For stone extraction as well as endoscopic evaluation of the intrapelvic lesion, percutaneous nephroscopy was performed. A small to large amount of tissue-like white debris in sheets characteristic of leukoplakia was found in the renal pelvis with stones embedded in it and was removed directly by forceps or suction and then by irrigating with saline. We propose that 1) the endourological approach should be recommended for patients with renal pelvic lesions associated with longstanding renal stones or for patients who show difficulty in passing stone fragments after ESWL and 2) this entity of leukoplakia should be kept in mind for the differential diagnosis of renal pelvic lesions associated with renal stones.

**KEY WORDS:** leukoplakia, renal pelvis, stone, endoscopy

### INTRODUCTION

Leukoplakia of the upper and lower urinary tract has been known for a long time. Usually it is defined as a cornification of a normally noncornifying membrane (1) and characterized microscopically as squamous metaplasia of the transitional epithelium and keratinization frequently associated with desquamation (2). However, its pathogenesis, pathological criteria for diagnosis, natural history, malignant potential, and treatment continue to be enigmatic (1–3). Although historically the term "cholesteatoma" was also used for similar conditions, the terminology has been somewhat confusing. Some authors have separated leukoplakia from cholesteatoma and defined them as two different entities (4,5). However, as proposed by Hertle and Androulakakis (2) leukoplakia-cholesteatoma should be considered as describing the same entity that can be expressed appropriately together under the term "keratinizing desquamative squamous metaplasia" (KDSM).

Address for correspondence: K. Nonomura, Department of Urology, Hokkaido University School of Medicine, Sapporo 060, Japan.

Regarding the management of leukoplakia-cholesteatoma, a radical operation has been the policy in the majority of surgically treated cases, the basic reasons being the fear of the development of cancer and the recurrence of the lesion (5,6). Recently conservative surgical management (kidney-preserving surgical management), sometimes combined with nonsurgical therapy, has been recommended for urothelial leukoplakia, since there is no evidence that it is a premalignant lesion nor necessarily a recurrent condition (7–9). Herein we present our experience of endourological evaluation and management of leukoplakia of the renal pelvis and discuss the importance of endoscopy, which should be integrated in the diagnosis and therapy for the intrapelvic lesion.

### CASE PRESENTATION

### Case 1

A 76-year-old man with a left renal stone was seen at our hospital. Three years ago he had undergone percutaneous nephrolithotripsy (PNL) at another hospital for a right renal pelvic stone that caused right flank pain and febrile urinary tract infection. At that time the left renal stone had been pointed out. After admission, routine laboratory studies, including kidney and liver function were performed and results were normal. Bacterial urine culture was positive (*Serratia marcescens*), and urine cytology was negative. Analysis of 24-hour urine samples revealed that urinary excretion of calcium, phosphate, and uric acid was within the normal range. On excretory urography [intravenous pyelogram (IVP)], a stringy radiolucent filling defect was documented in the left renal pelvis (Fig. 1), while the right kidney was normal. A computed tomography (CT) scan showed tissue with a honeycomb-like appearance in the left renal pelvis with a stone embedded in it (Fig. 2), indicating its different nature from a urothelial tumor.

For stone extraction as well as endoscopic evaluation of the intrapelvic lesion, percutaneous nephroscopy was performed using a Storz nephroscope. A large amount of tissue-like white debris in sheets was found in the renal pelvis, and a stone was embedded in the white debris. The stone was extracted with an ultrasonic probe, and the white debris was extirpated endourologically by suction. The resected material consisted of slender strips and sheets of mature, stratified squamous epithelium with prominent

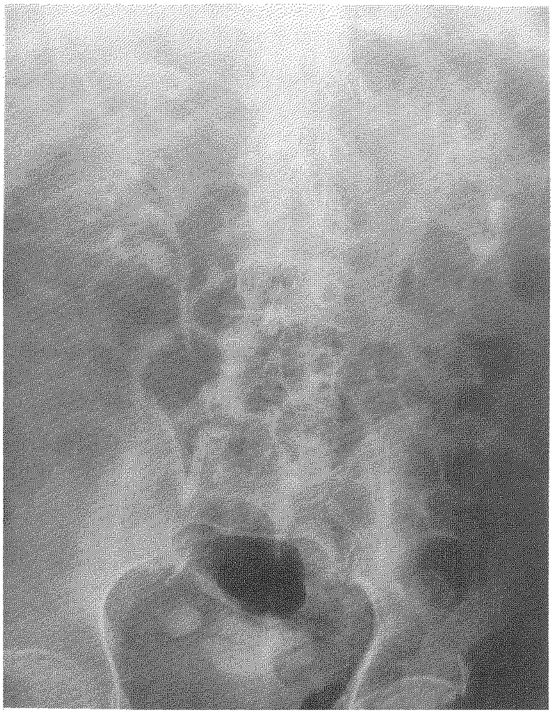

**Figure 1** IVP in Case 1. A stringy radiolucent filling defect was documented in the left renal pelvis.

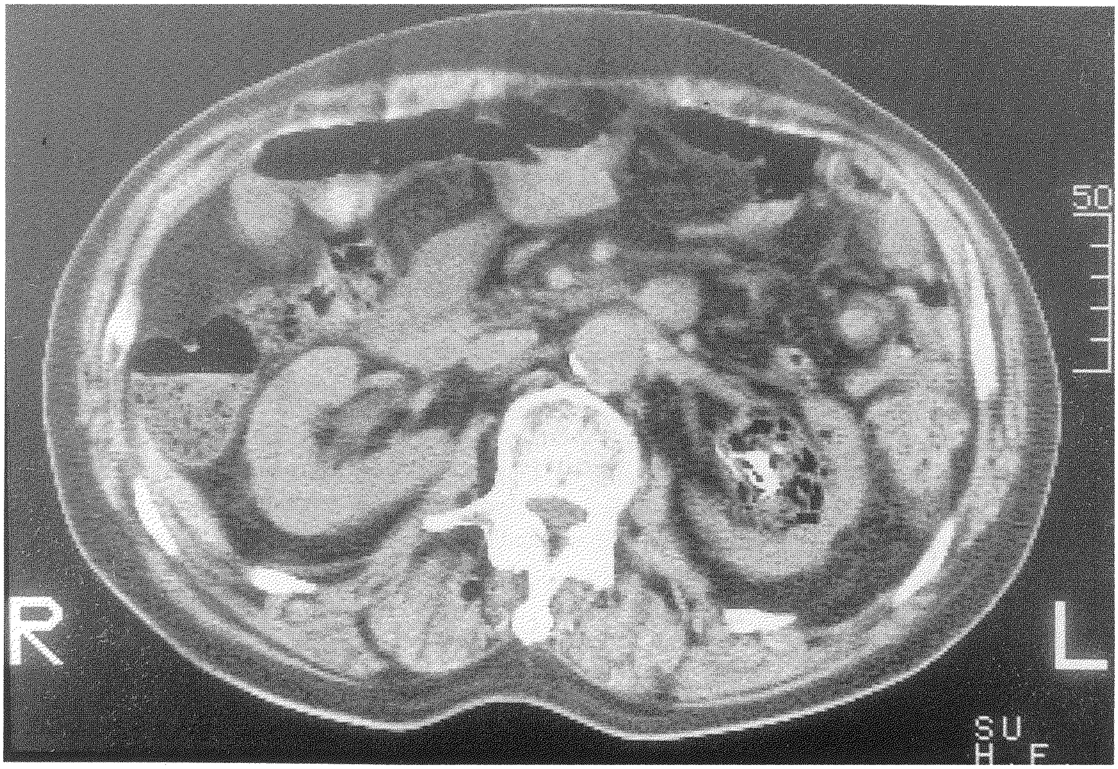

**Figure 2** CT scan in Case 1. Honeycomb-like tissue was noted in the left renal pelvis with stone embedded in it.

keratinization (Fig. 3). The pathological diagnosis was leukoplakia of the renal pelvis, and no malignant change was documented histologically. The stone was composed exclusively of calcium phosphate.

A postoperative CT scan revealed no residual stone and debris. However, a follow-up IVP performed about 20 months after the initial endourological treatment demonstrated the recurrence of the same lesion as well as a small stone in the left renal pelvis, and the second endourological procedure was performed in the same manner. Further follow-up was not available.

### Case 2

A 64-year-old woman was admitted to our hospital because of left renal colic. Five years ago, she had undergone extracorporeal shock wave lithotripsy (ESWL) for a left renal stone at another hospital, accompanied by residual stone. However, she had refused further treatment because of lack of symptoms. After admission, routine laboratory studies were performed and results were normal. Bacterial urine culture and urine cytology were both negative. Plain radiography revealed a left renal pelvic stone displaced inferiorly and multiple calyceal stones. IVP showed hydrocalyces and a radiolucent mass in the left renal pelvis. Retrograde pyelography clearly demonstrated multiple filling defects in the renal pelvis and middle calyces (Fig. 4A). For stone extraction as well as endoscopic evaluation of the intrapelvic lesion, percutaneous nephroscopy was performed. A large amount of white debris characteristic of leukoplakia was found in the renal pelvis with a stone embedded in it and extirpated by forceps or suction (Fig. 5). Histologically the white debris consisted of sheets of stratified squamous epithelium with prominent keratinization and was proved to be leukoplakia. For the next 2 weeks, the left renal pelvis was irrigated through a nephrostomy by saline, and then endoscopic evaluation was again performed. Keratinized white materials were found in some parts of the renal pelvis, but the proportion of mucosa with normal appearance increased dramatically. Residual white debris and a small stone were removed during this second procedure.

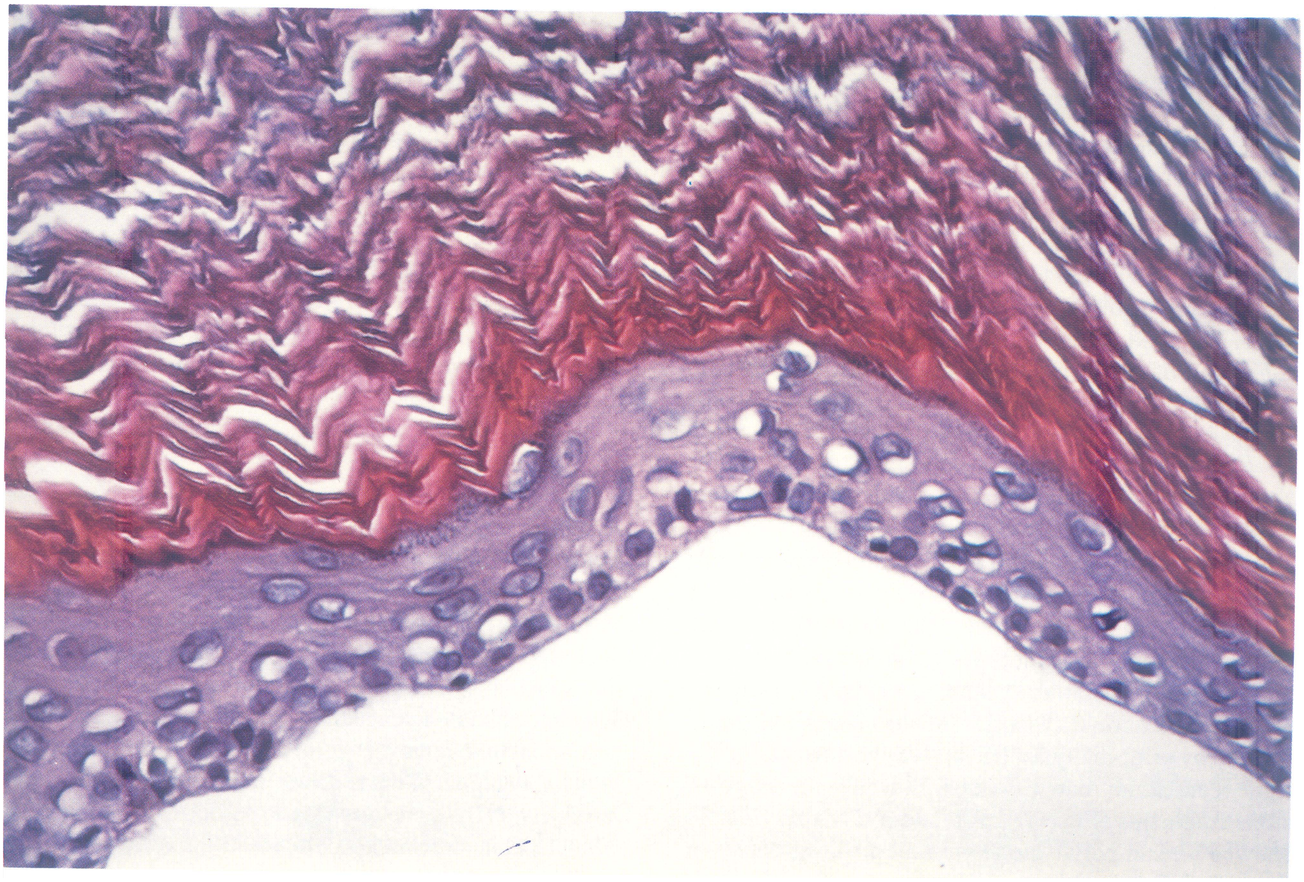

**Figure 3** Histological appearance of leukoplakia in Case 1. Stratified squamous epithelium with prominent keratinization was noted.

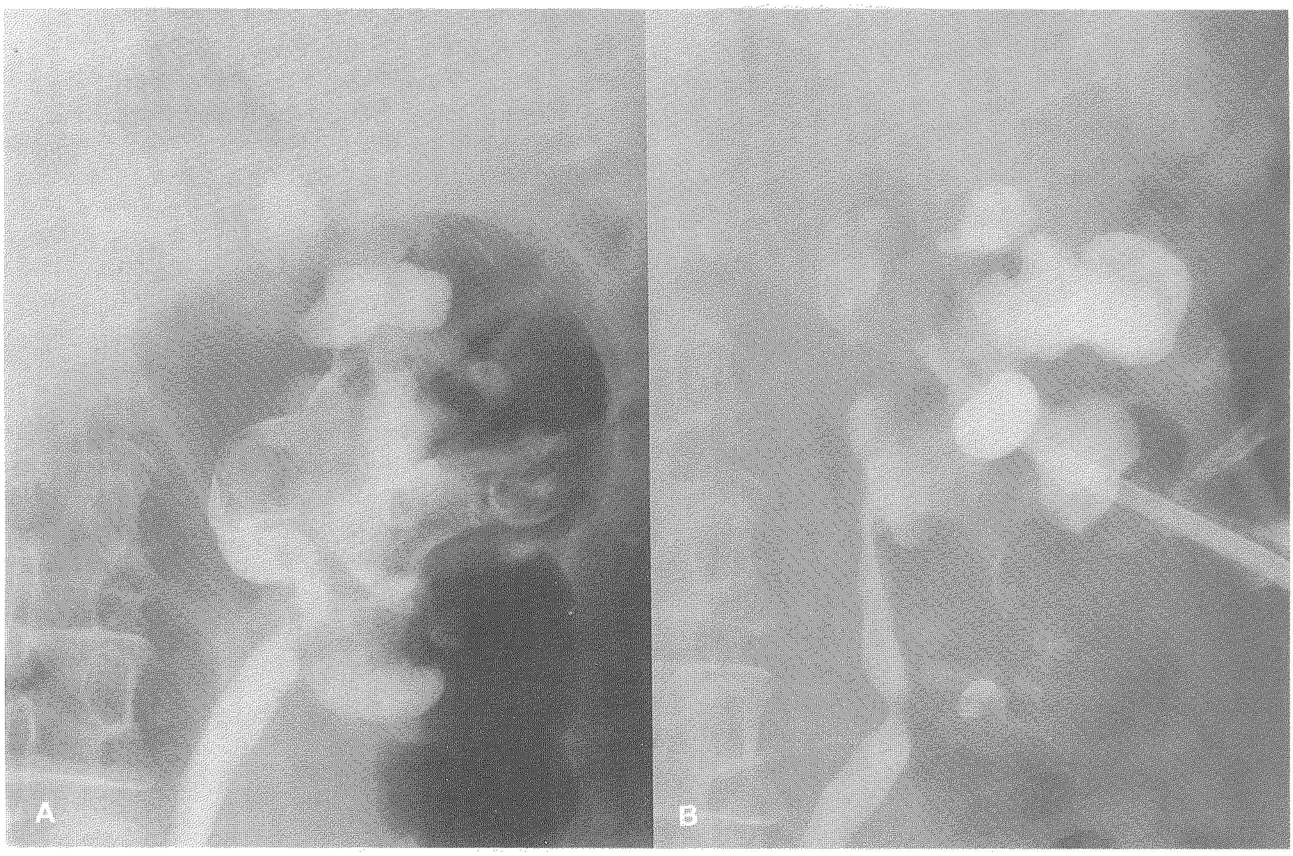

**Figure 4** A. Retrograde pyelography in Case 2, demonstrating multiple filling defects in the renal pelvis and middle calyces. B. Postoperative antegrade pyelography in Case 2, showing no filling defect in the left renal pelvis.

Postoperative antegrade pyelography through the nephrostomy showed no filling defect in the left renal pelvis and good urine drainage (Fig. 4B). The stone was composed of calcium phosphate and calcium oxalate. Recurrence of leukoplakia has not been documented during the following 2 years.

### Case 3

A 48-year-old man was referred to our hospital for a right renal stone (Fig. 6A). The right renal stone had been pointed out 4 years ago when he had undergone left nephrectomy elsewhere for tuberculosis. After admission, routine laboratory studies were performed and results were normal. Urinary infection was not documented by urine analysis. ESWL was performed for the right renal stone, but was not followed by passage of stone fragments (Fig. 6B). IVP and a CT scan (Fig. 7) showed a renal pelvic stone without a filling defect in the pelvis. For stone extraction, we performed PNL 10 days after ESWL. Stone fragments were closely associated with the renal pelvic mucosa. A small amount of white debris was found in the renal pelvis, which was proved histologically to be leukoplakia. No recurrence has been observed radiographically in the 2nd year after the operation.

### Case 4

A 56-year-old woman was referred to our hospital for left staghorn calculi (Fig. 8) that caused left flank pain. Urine analysis revealed severe hematopyuria. Bacterial urine culture was negative. Deteriorated split renal function of the left kidney was documented by renal dynamic scintigraphy using $^{99m}$Tc-diethylenetriamine pentaacetic acid. We performed PNL, expecting recovery of left renal function after stone removal.

A large amount of white debris in sheets was found in the renal pelvis. The stone was closely associated and mingled with the white debris and could not be extracted easily.

Left renal function did not recover after PNL. Furthermore, nephrostomy drainage was impaired be-

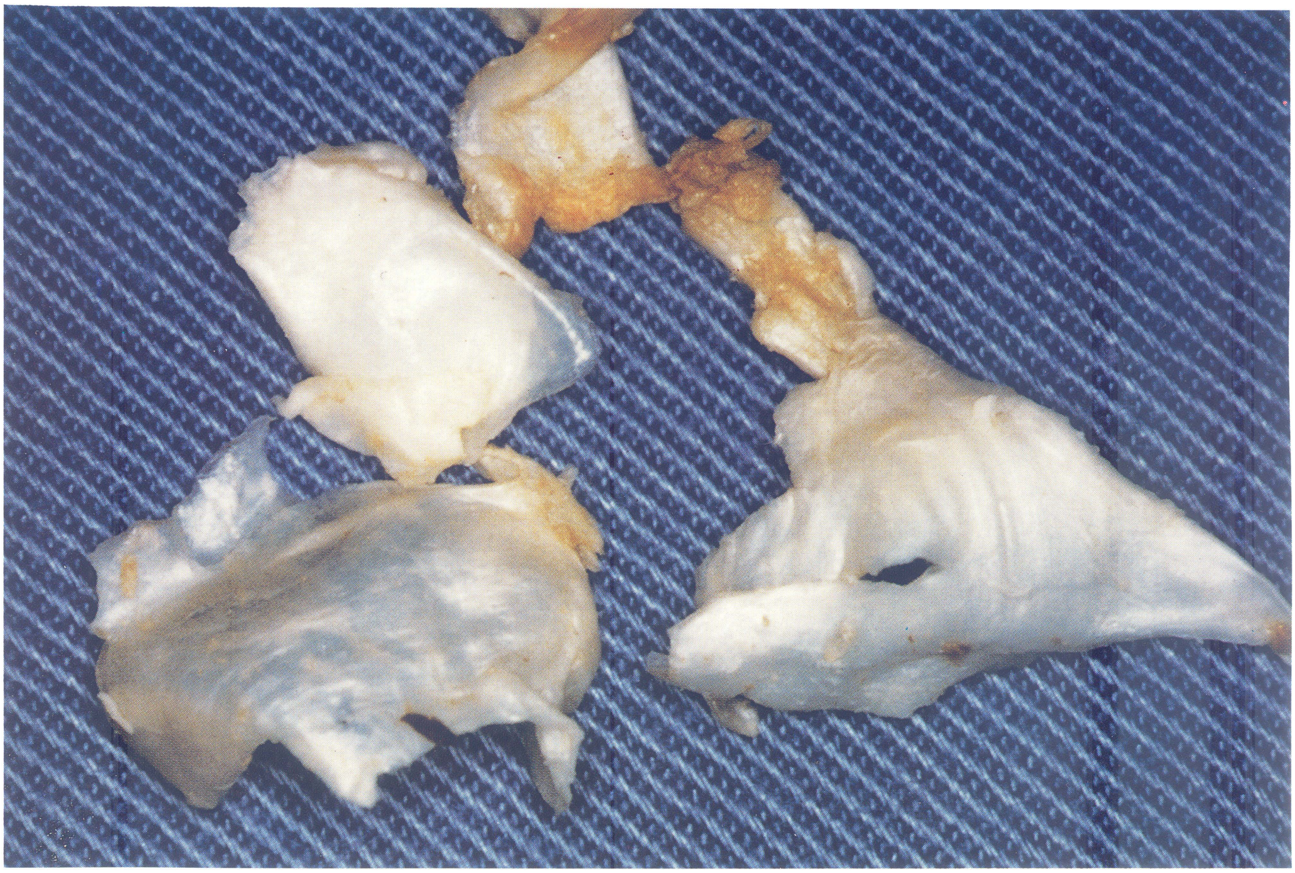

**Figure 5** Appearance of extirpated white debris in Case 2.

cause of residual stone and debris, so a left nephrectomy was performed 2 weeks after PNL. Histologically the white debris was proved to be leukoplakia.

### DISCUSSION

Squamous metaplasia of the transitional epithelium, keratinization, and subsequent desquamation of the keratinized layers (KDSM) constitute standard pathological features of leukoplakia and cholesteatoma (2). However, the pathogenetic relationship of squamous metaplasia, leukoplakia, cholesteatoma, and squamous cell carcinoma has been somewhat confusing, and some investigators insisted that the microscopic features of leukoplakia should include keratinizing squamous metaplasia with atypia (6,10) or dysplasia (1) of squamous epithelial layers. There also have been some reports describing the concept that leukoplakia is a premalignant disease because of the association of leukoplakia and cancer of the urinary tract (1,11). Kutzmann (12) suggests a simultaneous association of leukoplakia with malignancy of the upper urinary tract in about 12% of patients. However, the mere coincidence of leukoplakia and squamous cell carcinoma does not prove an etiological relationship between them. On the contrary, not a single case of leukoplakia-cholesteatoma in the upper urinary tract showing progression to squamous cell carcinoma has been documented to date, although a few cases of leukoplakia of the upper urinary tract with subsequent development of bladder carcinoma have been reported (1). Thus, we believe that leukoplakia-cholesteatoma in the upper urinary tract is a benign lesion on histological grounds.

Leukoplakia of the urinary tract occurs about three times as often in the bladder as in the renal pelvis and ureter (1). Based on a comprehensive study of 80 cases published in the literature, Hertle and Androulakakis (2) analyzed the pathological conditions associated with leukoplakia of the upper urinary tract. They found that recurrent urinary infections (21%), urolithiasis (19%), and tuberculosis (11%) were frequently associated with leukoplakia of the upper urinary tract, while no associated pathological con-

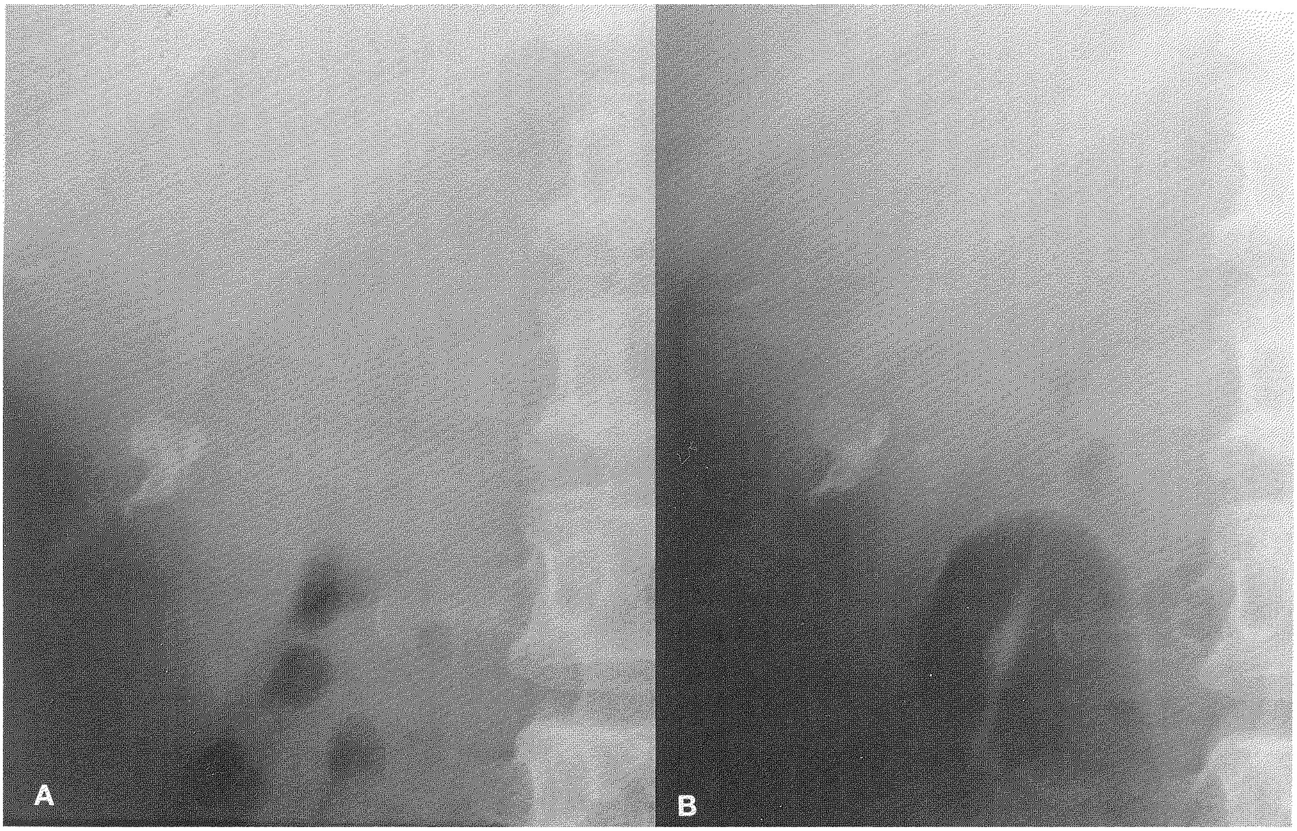

**Figure 6** Plain radiography in Case 3. A. Before ESWL. B. After ESWL. The stone shadow was not significantly changed after ESWL.

ditions were present in 40%. All of our patients had longstanding renal stones. Two theories have been proposed for the etiology of urothelial leukoplakia: 1) embryologically displaced cells of the ectodermal layer and 2) transformation or metaplasia of preexisting transitional epithelium either spontaneously or in response to certain noxious stimuli (1–3,8). Mueller et al. (8) described three women in one family suffering from longstanding irritation of the urinary tract who had systemic urothelial leukoplakia without any underlying pathological condition of the urinary tract. They supported the former theory by demonstrating ultrastructurally special cells in the basal layer of the cornified squamous epithelium of their patients that was similar to Merkel's cell of the skin. Although the exact etiology of urothelial leukoplakia is uncertain and may vary in individual patients, it is most important to keep in mind this entity of leukoplakia when we examine patients with recurrent urinary infections, urolithiasis, or longstanding irritative symptoms of the urinary tract.

Although the diagnosis of leukoplakia of the urinary tract could be made easily from a combination of the past history of recurrent urinary tract infection associated with calculous disease, the passage of white debris in the urine, and the radiological appearance of stringy radiolucent filling defects in the renal pelvis (13), it might be difficult in some cases without the typical findings of leukoplakia, resulting in unnecessary nephrectomy because of suspected urothelial tumor (14,15). If patients have a long history of recurrent attacks of flank pain with urinary tract infection or a renal stone associated with the passage of white debris (desquamated keratinized cells) in the urine and radiological examinations reveal stringy radiolucent filling defects in the renal pelvis, leukoplakia should be strongly suspected. None of our patients experienced the passage of white debris in the urine. The diagnosis of leukoplakia was first made by endoscopy (nephroscopy), which was confirmed by histological examinations.

With the advent of excellent optical equipment and a safe percutaneous puncture technique for the renal pelvis, renal pelvic lesions with or without a renal stone can be approached endoscopically either by the transurethral ureterorenoscope or percutaneous nephroscope. More recently, with the introduction of endourological methods,

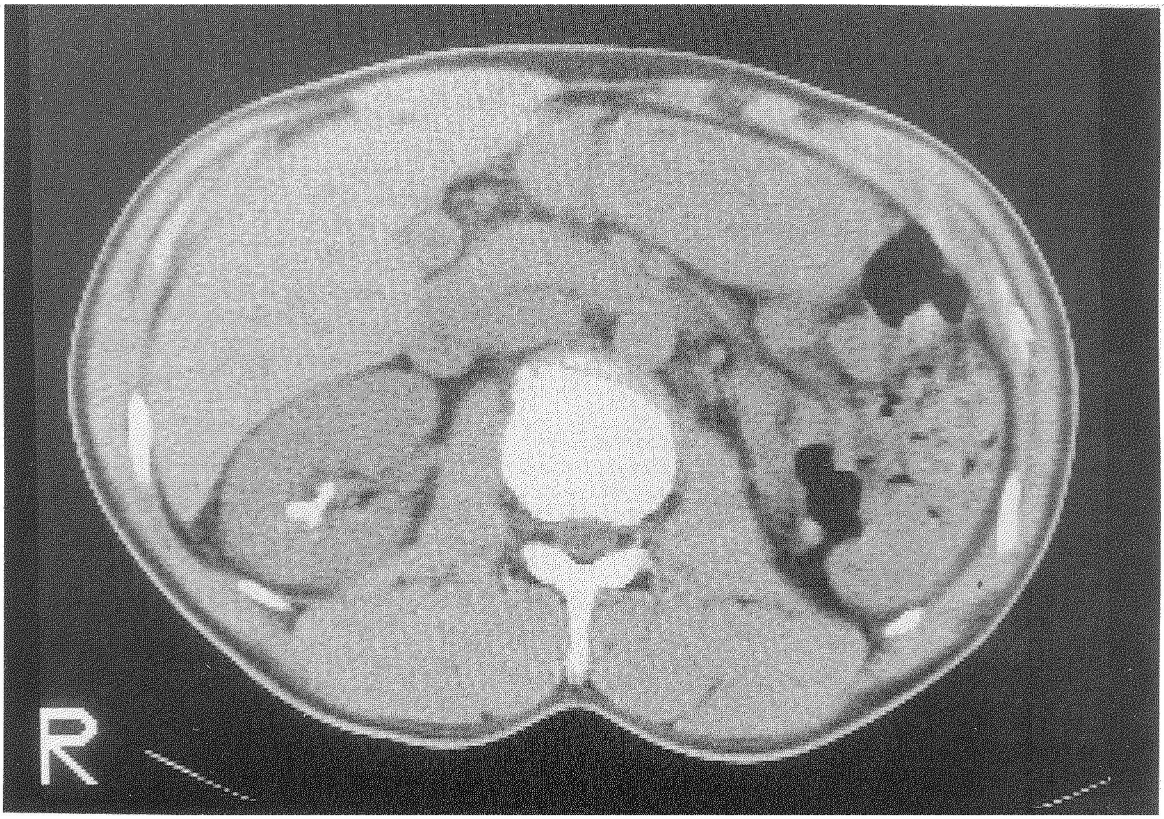

**Figure 7** CT scan after ESWL in Case 3. No intrapelvic lesion other than the stone was not noted.

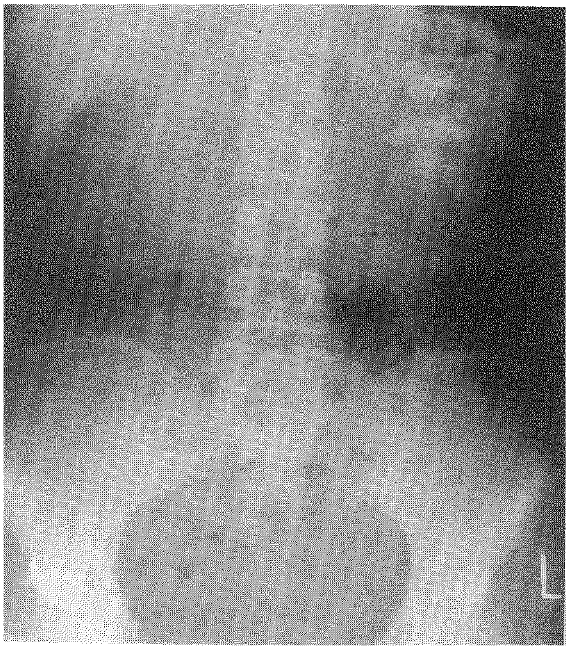

**Figure 8** Plain radiography in Case 4, showing staghorn calculi in the left kidney.

percutaneous renal surgery (endorenal surgery) has evolved as a parenchymal-sparing conservative surgery for renal stones and benign intrapelvic lesions or abnormalities such as inflammatory polyp or calyceal diverticulum (16). Furthermore, some investigators suggest that selected cases of renal pelvic transitional cell carcinoma might be amenable to percutaneous endourological resection (17,18). According to these trends in endourology, we have been actively performing percutaneous endourological procedures for renal pelvic lesions associated with longstanding renal stones or for patients who show difficulty in passing stone fragments after ESWL.

### REFERENCES

1. Benson RC, Jr, Swanson SK, Farrow GM. Relationship of leukoplakia to urothelial malignancy. J Urol 1984;131:507–511.
2. Hertle L, Androulakakis P. Keratinizing desquamative squamous metaplasia of the upper urinary tract: Leukoplakia-cholesteatoma. J Urol 1982;127:631–635.
3. Reece RW, Koontz WW, Jr. Leukoplakia of the urinary tract: A review. J Urol 1975;114:165–171.
4. Ross RR, Jr, Lewis HY, Brough AJ. Renal cholesteatoma in a child. J Urol 1970;104:184–188.

5. Weitzner S. Cholesteatoma of the calix. J Urol 1972;108:365–367.
6. Sarlis JN, Malakates SK, Papadimitriou D. Cholesteatoma of the renal pelvis. J Urol 1977;118:468–469.
7. Taguchi Y, Kotha V, Tomka B, et al. Conserving nephrons in cholesteatoma. J Urol 1980;123:258–260.
8. Mueller SC, Thueroff JW, Rumpelt HJ. Urothelial leukoplakia: Aspects of etiology and therapy. J Urol 1987;137:979–983.
9. Lupovitch A, Domzalski H, Tippins R. Cholesteatoma of the renal pelvis: A case with long-term followup. J Urol 1988;140:360–361.
10. Myrvold H, Fritjofsson A, Magnusson P. Cholesteatoma of the renal pelvis. Scand J Urol Nephrol 1974;8:69–72.
11. Fischelovitch J, Avidor I. Leukoplakia of renal pelvis. Br J Urol 1976;48:376.
12. Kutzmann AA. Squamous cell carcinoma of the renal pelvis: With special consideration as to etiology. J Urol 1938;39:487–505.
13. Freedberg LE, Bloustein PA, Stables DP, et al. Cholesteatoma of renal pelvis. Urology 1977;10:263–265.
14. Naumann HN, Sabatini SA. Cholesteatoma of kidney simulating squamous cell carcinoma. J Urol 1953;69:467–473.
15. Shrader DA, Bergreen PW. Cholesteatoma of ureter masquerading as ureteral tumor. Urology 1977;9:556–557.
16. Badlani G, Orihuela E, Smith AD. Percutaneous renal surgery (endorenal surgery). In: Smith AD, Castaneda-Zuniga WR, Bronson JG, eds. Endourology. principles and practice. New York: Thieme, 1986.
17. Streem SB, Pontes EJ. Percutaneous management of upper tract transitional cell carcinoma. J Urol 1986;135:773–775.
18. Smith AD, Orihuela E, Crowley AR. Percutaneous management of renal pelvic tumors: A treatment option in selected cases. J Urol 1987;137:852–856.

